# Network Pharmacology: A New Approach for Chinese Herbal Medicine Research

**DOI:** 10.1155/2013/621423

**Published:** 2013-05-20

**Authors:** Gui-biao Zhang, Qing-ya Li, Qi-long Chen, Shi-bing Su

**Affiliations:** ^1^Research Center for Traditional Chinese Medicine Complexity System, Shanghai University of Traditional Chinese Medicine, Shanghai 201203, China; ^2^Henan University of TCM, Zhengzhou 450008, China

## Abstract

The dominant paradigm of “one gene, one target, one disease” has influenced many aspects of drug discovery strategy. However, in recent years, it has been appreciated that many effective drugs act on multiple targets rather than a single one. As an integrated multidisciplinary concept, network pharmacology, which is based on system biology and polypharmacology, affords a novel network mode of “multiple targets, multiple effects, complex diseases” and replaces the “magic bullets” by “magic shotguns.” Chinese herbal medicine (CHM) has been recognized as one of the most important strategies in complementary and alternative medicine. Though CHM has been practiced for a very long time, its effectiveness and beneficial contribution to public health has not been fully recognized. Also, the knowledge on the mechanisms of CHM formulas is scarce. In the present review, the concept and significance of network pharmacology is briefly introduced. The application and potential role of network pharmacology in the CHM fields is also discussed, such as data collection, target prediction, network visualization, multicomponent interaction, and network toxicology. Furthermore, the developing tendency of network pharmacology is also summarized, and its role in CHM research is discussed.

## 1. Introduction

Over the past decades, drug discovery has followed the dominant paradigm of the “one gene, one drug, one disease” and mainly focused on designing exquisitely selective ligands which could avoid side effects [1]. However, owing to the lack of efficacy and safety, the clinical attrition rate of new drug candidates reached up to 30% [2]. Moreover, the large-scale functional genomics studies have revealed that many single-gene knockouts exhibit little effect on the phenotype [3], and only 34% of single-gene knockout resulted in sickness or lethality [4]. Systems biology is a recent trend in bioscience research which focuses on the complex interactions in biological systems from a holistic perspective, rather than altering the single molecular component [5, 6]. Network pharmacology [7, 8], a system biology-based methodology, replaces the corollary of rational drug design of “magic bullets” by the search for multitarget drugs that act on biological networks as “magic shotguns” [9]. Network biology analysis has also revealed that the deletion of individual nodes has little effect on the disease networks [10]. The increased understanding of the role of network biology systems challenges the dominant assumption of single-target drug discovery [11, 12]. Chinese herbal medicines (CHM) include natural medicines that were discovered by the ancient Chinese and evolved through at least 3000 years of uninterrupted clinical practice. Generally, CHM cures diseases by the synergistic effects of multiple compounds and herbal formula, which is mainly based on the integrative and holistic ways [13]. However, with the growing popularity and great promise of CHM, the ever-increasing demand for illuminating pharmacological mechanisms, potential drug efficacy, and clinical toxicity are major issues that need to be addressed. As a methodology and technology, network pharmacology offers a new approach to integrate the notion of drug discovery based on comprehensive research and synthetic assessment. Obviously, this principle coincides with the characteristics of syndrome differentiation by traditional Chinese medicine (TCM) and holistic view of CHM treatment [14].

 In this review, the concept and significance of network pharmacology is briefly introduced. Its application and potential role in CHM research is also summarized.

## 2. Concept and Significance of Network Pharmacology

With the rapid growth of available biomedical data in the postgenomic era, systems biology and polypharmacology have provided fresh insight into the drug discovery [15, 16]. The computational biology provides profitable approach to address the scientific suspense through efficacious modeling and theoretical exploration. In 2007, Hopkins created a novel concept of network pharmacology, which is built on the fundamental concept that many effective drugs in therapeutic areas act on multiple rather than single targets [7, 9]. Network pharmacology can be reconstructed with molecular networks that integrate multidisciplinary concepts including biochemical, bioinformatics, and systems biology [8]. It affords a rewarding assistance to forecast the off-target effects at a higher efficiency, which could improve the potency for drug discovery through a novel network mode of “multiple targets, multiple effects, complex diseases” [17].

The advantages of network pharmacology include the following: regulation of the signaling pathway with multiple channels, increase in drug efficacy, reduction of side effects, increase in the success rate of clinical trials, and decrease in the costs of drug discovery. Many complex diseases involve the interactions of multiple genes and functional proteins [18]. Network pharmacology models aim at addressing questions such as how and where in the disease network one target inhibits or activates the disease phenotypes. This ideally leads to therapies that are less vulnerable to drug resistance and lesser side effects by means of attacking the disease network at the systems level through synergistic and lethal interactions. The drug discovery strategies thereby should explore the regulated pathology network and reduce the typically high attrition rates in the disease networks [19]. Many studies have reported the interesting biological findings from these networks, and more than 40% of the drug-activated targets were discovered based on the disease gene networks by meta-analysis [20], which were associated with a number of diseases [21]. Therefore, network pharmacology can assist in systematic characterization of drug targets, thereby helping to decrease the high failure rates in discovery projects.

## 3. Research Approaches of Network Pharmacology

Network pharmacology can make an impact at two main approaches in the drug development process. One is to establish a pragmatic network model and predict the drug target based on public databases or available data of earlier researches. Subsequently, the mechanism of functional drug should be explored for the network equilibrium principle. Based on this approach, Gu et al. calculated the effect of Rheidin A and C and Sennoside C, which was the first report on multiple component drugs for type II diabetes [22]. Yan et al. also predicted the new pharmacological action of Ephedra Decoction using virtual screening and network forecast techniques [23].

 The other approach is to reconstruct a “drug target disease” network prediction model using the high-throughput screen (HTS) technology and bioinformatics methods. In this approach, the mechanism of drugs in the biological network was analyzed by comparing the interaction between the drug and the model. Many examples on the application of network pharmacology in drug discovery have been reported in the literature. Li et al. [24] used Liuwei Dihuang pill (a CHM formula) to predict the suitable network targets in disease treatment and found that the multilayer networks may underlie the combined mechanisms of herbal formula. Furthermore, nine components were screened in Fufang Danshen formula based on network pharmacology, which could modulate 42 cardiovascular-associated genes [25]. Additionally, by integrating the above research approaches, it was demonstrated that salvianolic acid B was compatible and feasible for cardiovascular disease treatment [26].

## 4. Process in Network Pharmacology Research

### 4.1. Data Collection and Validation

Network pharmacology takes into account the aforementioned principles to optimize the efficacy and safety of a candidate drug and their potent combinations. These also represent the two important steps in any experimental study. The first step of network pharmacology is the selection of original data from the experiments to build a biological network. The second is the experimental validation for the predicted network model. The validated data can be quantified using many different integrated methods including genomics, proteomics, metabolomics, and HTS/high-content screening (HCS) technologies [27].

HTS/HCS technologies can rapidly detect millions of data samples, identify substances, and modulate a particular molecular pathway or alter the phenotype of a cell [28, 29]. These have many desirable features such as homogeneous, multidimensional phenotypic detection, real-time, dynamic monitoring, and visualization. Moreover, this dual high-throughput technology also can collect network data from the experiments and validate the network model. For instance, Fakhari and Dittmer created the polymerase chain reaction (PCR) chip technology to detect the gene expression [30]. The results demonstrated that the technology was convenient for the high-throughput studies.

Molecular interaction validation technology is another tool which validates the approach for network pharmacology, reveals the drug activity mechanisms, and verifies the drug network or predicted model. It can help researchers to discover the relationship between the drug and the macromolecules, and it mainly includes surface plasmon resonance (SPR) [31] and biolayer interferometry (BLI) technologies [32]. All of these techniques involve high-throughput, high-precision, label-free, and real-time detection.

### 4.2. Network Analysis and Visualization

Network analysis focuses the on established network using related technology and extracts useful information which is convenient for further studies [33]. Three types of network analysis are available. The first one involves the calculation of the optimal topological structure and statistical properties of network after the extraction of specific network data [34], which is conserved as the hidden information in the network maximally. Secondly, the generation and comparison of random networks is used to check the reliability of the existing network by inducing acceptable modulation [35]. At last, the hierarchical clustering of the network is performed [36], algorithm is applied to predigest the complicated network, and potential information in the network is anticipated.

 Network visualization is applied to extract the interaction information from interassociation data and switch them into a visual network using visualization tools [33]. This process contains two steps: (1) enriching network attributes, adding network nodes, and increasing connection power of network; (2) describing the network and taking abundant instrument to describe the architectural feature that clearly and intuitively represents the network. At present, most visualization of network pharmacology is through professional tools such as Cytoscape [37], GUESS [38], and Pajek [39]. Brief information of network pharmacological technologies and tools is shown in Table 1.

## 5. Application of Network Pharmacology in CHM Research

**Figure 1:**
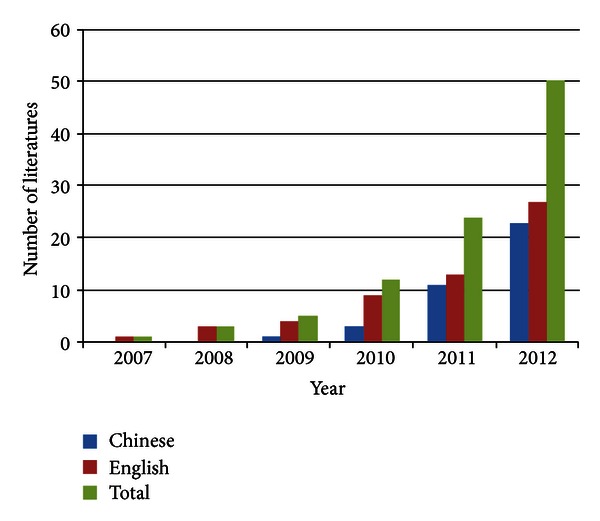
Developing tendency of network pharmacology study. The publications of network pharmacology study in Web of Knowledge, PubMed, and CNKI databases from 2007 to 2012. All results were screened in manual way.

CHM, the ancient treatment methodology popular in China and surrounding areas, has been recognized as a pharmaceutical area of TCM and holds promise for preventing diseases in a holistic way [40, 41]. In a long period of clinical practice, it is known for its effectiveness and beneficial contribution to public health and disease control. However, the pharmacological mechanisms of CHM have not been fully established. With increasing knowledge of the network of genes and molecular interactions, the researchers adopt network pharmacology for their drug research and development.  Figure 1 shows the developing tendency of network pharmacological studies from the data available in Web of Knowledge, PubMed, and China National Knowledge Infrastructure (CNKI) databases from 2007 to 2012. The applications of network pharmacology in CHM were systematically summarized to demonstrate the significant value in this area of research.

### 5.1. Construction and Application of CHM Database

Building a CHM database is critical for a network pharmacology study. Chen et al. [42] constructed the TCM-ID database including TCM prescriptions, herbal ingredients, and 3D structure of herbal ingredients. It was mainly used for illustrating the mechanism of the effects of herbal ingredients. Ye et al. [43] integrated the text-mining technology, a strict artificial audit and annotation process, and obtained the protein targets of Chinese medicine by determining the effective components based on the mass literature from PubMed. Subsequently, the Herbal Ingredients' Targets Database provided the integrated information for the Chinese herbal active ingredient protein target and offered platform to analyze the similarity between the compound formula and protein sequence. 

 Furthermore, many other TCM databases have been established for network pharmacology research, such as the TCMGeneDIT database [44] and TCM Database@Taiwan [45]. The former mainly focuses on TCM-related gene and disease information, and the latter is applied to CHM screening. In addition, the disease-drug-target databases such as SuperTarget, Matador [46], DrugBank [47], and Therapeutic Target Database [48] are also used for drug-target research on herbal compounds.

### 5.2. Predictions of CHM Drug Target

Relevant technology can be used to screen the effective herbal substances and discover the drug target. Such technology could also provide theoretical support for detecting new pharmacological effects of Chinese compound formula. Li [49] proposed a methodology termed “network target,” which is used to reveal the interactions between herbal compounds/formulas and complex syndrome systems based on network pharmacology and systems biology. Wu et al. [50] predicted the multitarget of *Aconiti Lateralis Radix Praeparata's* multi-compound based on the drug-target data and random forest algorithm. They found that each compound was correlated with 16.3 targets, whereas each target was related to 4.77 compounds. This study reflects the notion of “multi-compound and multitarget” in CHM towards drug discovery. 

 Yu et al. [51] predicted the new pharmacological effects (antihyperglycemic and antihyperlipidemic) of Fuzheng Huayu Capsule using high-throughput technology and Connectivity Map databases [52]. Using the chemical structure of compounds, target identification, and enrichment analysis, Zhu and Yao [53] successfully predicted the molecular targets of Xiaochaihu Decoction. They found that the potential targets of the 21 compounds mainly involved metabolic, inflammation, and poison degradation processes. Zhang et al. [54] created a new algorithm to predict the molecular targets of rhein, which integrated the protein-protein interactions, pathways, genome expression, and the literature data mining. The results showed that three specific genes were relevant for drug targets, and their functions mainly involved cellular apoptosis, immunity, and transport.

### 5.3. Network Visualization of the CHM Literature Mining Researches

Essentially, the network visualization of the CHM literature examines the database to find modes or rules [55], detects the literature information, analyzes the selection data, and discovers the novel effects of CHM. For example, Li et al. [56] integrated the microarray and the literature database to design a literature mining and microarray analysis system, which was used to construct biological networks and reveal the related interactions. 

 Wu et al. [57] collected slices information of TCM from the Chinese Pharmacopoeia by text mining and constructed the TCM slices-symptom network. They discovered 3016 pairs of TCM slice-symptom correlations. Each TCM slice was correlated with 4.67 symptoms, and each symptom was related to 7.47 TCM slices. Furthermore, the network analysis also indicated that network pharmacology approaches could be used to forecast and discover unknown or new effects and channel tropisms of TCM slices. Additionally, Zhang et al. [58] reconstructed three Bayesian belief networks [59] for bitter, sweet, and pungent flavors, which was based on the modern pharmacology of TCM slices and clinical data. The results suggested that the Bayesian belief network could be used to predict the flavors of Chinese medicinal components and perform further studies of drug properties and drug compatibility.

### 5.4. CHM Multicomponent and Formula Researches

In order to discover the relationship between Chinese herbal multicomponent and potential pharmacological function, Li et al. [60] applied a network target-based identification of multicomponent synergy (NIMS) algorithms to calculate the component of CHM and demonstrate the synergistic correlation of the multicomponent. The results proposed that the NIMS approach could be beneficial for analyzing the therapeutic effects of CHM multicomponent. In the Tougu Xiaotong capsule (TGXTC) study, Zheng et al. [61] analyzed 514 components using network pharmacology and computational pharmacological methods. By analyzing the network parameters of the TGXTC compound-target and drug-target networks, they revealed the molecular mechanism of multicomponent, multitarget, and multipathway in TGXTC.

 Zhu et al. [62] used molecular docking and complex analytical techniques to study the pharmacodynamic function of multicomponent in spleen-regulating heart-nourishing formula. They found that the scale-free feature and node attribute of networks clearly illuminated the pharmacological function of CHM multicomponent. Exhilaratingly, some recent studies revealed the mechanisms of multicomponent efficiency in CHM for the treatment of cardiovascular disease [63, 64] by systematic investigation, which have constructed “CHM components targets” networks based on chemical components, chemical structures, chemogenomics, and target predictors data.

### 5.5. CHM Network Toxicology Researches

Network toxicology is based on comprehension of “toxicity (side effects) gene target drug,” which utilizes the network analysis to speculate and estimate toxicity and side effects of drugs. It focuses on the toxic reaction of specific component in a complex system and provides assistance for drug safety evaluation and research. The study of Liu et al. [65] identified that the integrated high-throughput biochip technologies and drug-target network could afford significant values for activated ingredient screening, toxic components exclusion, and molecular mechanisms of CHM research. Fan et al. [66] used network pharmacology method to reconstruct the network model to describe the toxicological properties, which offered valuable information to identify the toxic substances and potential toxicity of known compounds in a complex system. Zhou et al. [67] analyzed the nephrotoxicity of aristolochic acid based on the metabolic network and established an integral and dynamical progression of drug toxicity method.

 In addition, the databases such as Comparative Toxicogenomics Database, National Toxicology Program, and Toxicology Data Network are widely used for network toxicology studies [68, 69]. Furthermore, the forecasting toxicity software such as Toxicity Prediction by Komputer-Assisted Technology, HazardExpert, DEREK, and Prediction System of Carcinogenic Toxicity [70] is other available tools for CHM network toxicology studies.

### 5.6. TCM Syndrome-Based Network Pharmacology Researches

The feature of TCM is based on the syndrome differentiation, which emphasizes the integrating disease and syndrome. Monarch, minister, assistant, and guide in TCM prescription contain many principles of system theory, and the aim of coordination and cooperation of several kinds of CHM is to regulate body functional imbalances and disorders. Therefore, network pharmacology could be used for TCM diagnosis based on “disease-syndrome-formula” model, which integrates the information of “disease-phenotype-gene-drug” and builds a “disease phenotype biological molecule” network [24]. In a study on patients with rheumatoid arthritis (RA), Niu et al. [71] revealed the molecular mechanism of “herbs-pattern correspondence” with heat pattern in TCM, which analyzed the drug-target molecular network. Four common canonical pathways were found to be involved in these: GM-CSF signaling, CLTA4 signaling in cytotoxic T lymphocytes, T-cell receptor signaling, and CD28 signaling in T-helper cells. These uniform pathways implicated that the “herbs-pattern correspondence” could more likely be associated with heat pattern of RA.

ZHENG is a complex concept in TCM. Li et al. [72] reconstructed the neuroendocrine-immune (NEI) network by systems biology approach combined with animal experiments. The results showed that the hormones were predominant in the Cold ZHENG network, whereas immune factors were predominant in the Hot ZHENG network. In particular, two networks were connected by neurotransmitters, which suggested that ZHENG might have a special molecular mechanism from the background of NEI study. Using the microarray samples of liver-gallbladder dampness-heat syndrome and liver depression and spleen deficiency syndrome in chronic hepatitis B and liver cirrhosis, Guo et al. [73] elucidated the molecular mechanisms of the same TCM syndrome for different diseases and different TCM syndrome for the same disease, which might be related to the G-protein-coupled receptor protein-signaling pathway. Moreover, Shi et al. [74] used a complex network and chi-squared automatic interaction detector decision tree to identify the core syndromes of TCM in coronary heart disease (CHD) and establish TCM syndrome identification modes of CHD based on the four diagnostic information and biological parameters.

## 6. Discussion and Conclusion

Although TCM has a long history of clinical practice in China, it is considered as a complementary and alternative medicine in the rest of the world. The biggest obstacles of CHM development are the multiple *in vivo* pathways of CHM metabolites from their multiple drug components, which are essential for their pharmacological actions. However, the knowledge on the effective mechanisms of CHM formulas is scarce. Moreover, the CHM mechanism is considered as the synergistic effect of active drug ingredients and results of complex biological interactions. Fortunately, as a novel approach, network pharmacology takes into account the multidisciplinary and cross-disciplinary fields to optimize the efficacy and safety of drug discovery. Hence, researchers started utilizing it to study the drug targets and efficacy of CHM [75]. Network pharmacology has become a helpful tool to understand the details of drug-target, especially for multiple drug components of CHM.

Generally, network pharmacology of CHM has been considered to contain static and dynamic configurations. The static configuration is called a network pharmacy metrology with chromatographic fingerprint, while the dynamic is called a network pharmacodynamics with chromatographic fingerprint (NPDCF). The key problem for CHM formula network pharmacology is how to confirm the parameters of NPDCF by network equilibrium constants [76]. For example, He et al. [77] analyzed the multiple drug components from the parameters of network pharmacokinetic model and found that the effects of CHM formulas were inhibited by pharmacokinetic and pharmacodynamic coefficients [78]. In the static multicomponent studies of CHM formula, Tal et al. [79] predicted the active ingredients and potential targets of Chinese herbal *Radix Curcumae* formula using network pharmacology, which was used for the treatment of cardiovascular disease. However, the network dynamics research of CHM formulas need more evidence.

**Figure 2:**
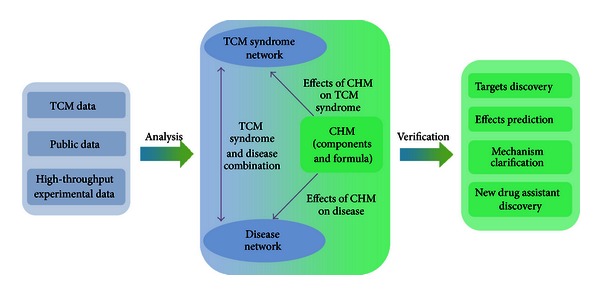
Network pharmacology approach for CHM research. For the discovery of CHM-derived targets, effect prediction, mechanism clarification, and new drug assistant discovery using network pharmacology approach. It analyzes the information from public data, high-throughput experimental data, and TCM data and constructs a “CHM-TCM syndrome disease” interaction network using technologies of network expansion, optimization, comparison, knockout, and addition. Finally, it carries out computational and experimental verifications.

The characteristics of TCM theory involve the consideration of organic wholeness and treatment based on TCM syndrome differentiation. A diagram is proposed to exhibit the research approach of network pharmacology for CHM (Figure 2). This approach is a combination from “disease-syndrome-CHM” model, which comprises the core values for reflecting disease and TCM syndrome as well as correlates with CHM, TCM syndrome, and multitarget effects. By integrating the chemical predictor, target predictor, and network building, a system of TCM was constructed. It systematically revealed the potential mechanisms of TCM [80]. The appropriate cellular and animal models are conducive to evaluate the effectiveness of TCM [81–83], which could be used to verify the results of network analysis and mutual authentication. However, the systemic characterization is still unclear for the drug-target correlation of CHM. Network pharmacology could be helpful to confirm the effective ingredients and promote drug discovery of CHM. 

Network pharmacology has become a helpful tool in understanding the fine details of drug-target interactions. Using network pharmacology to investigate CHM pharmacological effects and drug targets, attention should be paid to degree centrality [84], betweenness centrality [85], and bridging centrality [86]. Network-based tools for analyzing topology and especially dynamics have great potential to identify alternative targets for finding and developing multitarget drugs [87]. 

In summary, the advancements in systems biology and bioinformatics will make an operational shift from reductionism in favor of network pharmacology and will undoubtedly bring about a conceptual move in drug discovery and make a significant contribution to CHM modernization and globalization.

## Figures and Tables

**Table 1:** Brief introduction of network pharmacological technologies methods and tools.

Mainly experimental techniques and tools in network pharmacology			
Technique	Application fields	Advantage	Literatures

HTS/HCS	Massive data acquisition	Homogeneous, multidimensional phenotypic detection, dynamic real-time monitoring, and visualization	[28, 29]
PCR chip	Massive data acquisition	Dual high throughput, strong specificity, high sensitivity, and good repeatability	[30]
SPR	Massive data acquisition	No marks, high-throughput, high-precision, and real-time detection	[31, 32]
BLI	Massive data acquisition	No marks, high-throughput, high-precision, and real-time detection	[31, 32]
Cytoscape	Network visualization	Graphic operation, construct simple network, plugin support for analysis, and easy to use	[37]
GUESS	Network visualization	Graphic operation, command line, and script support for analysis	[38]
Pajek	Network visualization	Graphic operation, building large-scale network	[39]
Network topology information calculation	Network analysis	Classify and sequence the nodes, reflect hidden information	[34]
Random network creation and comparison	Network analysis	Verify reliability of existing network	[35]
Network layer and clustering	Network analysis	Simplify the complexity of network, find out potential information	[36]
